# Uptake and Diagnostic Yield of Chromosomal Microarray in an Australian Child Development Clinic

**DOI:** 10.3390/children1010021

**Published:** 2014-05-09

**Authors:** Dylan Mordaunt, Michael Gabbett, Melanie Waugh, Karen O’Brien, Helen Heussler

**Affiliations:** 1 Mater Children’s Hospital, Raymond Terrace, South Brisbane, QLD 4101, Australia; E-Mails: mlwgmo@gmail.com (M.W.); k.obrien@psy.uq.edu.au (K.B.); Honey.Heussler@mater.org.au (H.H.); 2 SA Pathology at the Women’s and Children’s Hospital, 72 King William Road, North Adelaide, SA 5006, Australia; 3 Genetic Health Queensland, Back Street, off Bramston Terrace, Herston, QLD 4029, Australia; E-Mail: michael.gabbett@health.qld.gov.au

**Keywords:** chromosomal microarray, autistic disorder, autism, Asperger syndrome, Rett syndrome, childhood disintegrative disorder, pervasive developmental disorder-not otherwise specified

## Abstract

Autism is an etiologically heterogeneous developmental disorder for which the range of genetic investigations has expanded considerably over the past decade. Introduction of chromosomal microarray (CMA) to clinical practice has expanded the range of conditions which pediatricians are able to detect. This study reviewed the utilization, yield and cost of genetic investigations in a sample of children with pervasive developmental disorders (PDD) in an Australian metropolitan child development service. Six hundred and ninety eight patients with PDD were identified from the clinic population. One hundred and ten (15.7%) of the clinic population had undergone investigation with chromosomal microarray, 140 (20.0%) with karyotype (KT), and 167 (23.9%) with Fragile X testing (FRGX). Twelve (10.9%) CMA findings were reported, of which seven (6.3%) were felt to be the likely cause of the child’s clinical features. Five (3.5%) KT findings were reported, of which four (2.9%) were felt to be the likely cause of the child’s clinical features. Two patients (1.2%) were identified with Fragile X expansions. One fifth of the clinic’s recent PDD population had undergone testing with CMA. CMA appears to have increased the diagnostic yield of the genetic investigation of autism, in line with internationally reported levels. Number needed to test (NNT) and cost per incremental diagnosis, were also in line with internationally reported levels.

## 1. Introduction

An increasing number of genetic and biochemical investigations have become accessible to pediatricians for the etiologic investigation of children with neurodevelopmental disorders. The introduction of chromosomal microarray to the Australian Government Medicare Benefits Scheme (MBS) for investigation of developmental delay in 2010 [1], represented a milestone in clinical genetic testing in Australia. However, there have been no Australasian guidelines advising on the recommended use of genetic investigations in autism [2].

International guidelines recommend specific approaches to the use of genetic investigation in autism [3], including the use of chromosomal microarray (CMA) [4] and screening for metabolic disorders as first-tier investigations.

These international guidelines have been largely based on studies performed in prospective cohorts, assessed in specialty clinic populations, rather than in community or hospital-based child development clinic populations [2,3,4,5,6,7,8,9,10,11]. There are no Australasian reports of the uptake and yield of CMA.

There are few publications about the influence of service context on the yield of testing strategies. There are few publications on the financial implications of genetic testing on health care funders and providers in Australia. Limited evidence suggests that patients referred to a child development service may have a lower pre-test probability of a positive genetic test with comparison to a clinical genetics service [12]. This further raises the question of generalizability of previously published reports to general and community pediatricians. More recent reports demonstrate an increased positive likelihood ratio of chromosomal microarray in the context of Autism combined with particular comorbidities, such as cognitive (intellectual) impairment [12,13] and epilepsy [13].

CMA has anecdotally become a first-tier investigation in both “syndromic”/“non-syndromic” and simplex/multiplex autism, and is a de facto standard of investigation of autism in our practice [14]. Our aim was to review our use of CMA, including the uptake and yield of CMA in our clinic, and therefore to evaluate the real-world utility of chromosomal microarray for the etiological investigation of autism. By yield, we mean the proportion of investigations in which a genetic variant is reported. We also aimed to develop a cost index by which to compare the relative financial value of the testing method used. This cost index may also serve as a means of comparison newer genomic investigations platforms in the future, as well as inclusion of changes in pricing.

## 2. Methods

We performed a retrospective audit of genetic testing of patients diagnosed with autism in our clinic. We prospectively obtained institutional ethics approval, protocol reference number 2012-62, 26 September 2012.

### 2.1. Study Sample

We identified eligible participants through a review of hospital billing records and included consecutive patients seen and diagnosed in our clinic with pervasive developmental disorder (PDD), according to the DSM-IV-TR [15,16]. Pathology investigation results were then obtained on these individuals by cross-referencing with our institution’s Pathology Information Management System.

### 2.2. Clinical Assessment and Diagnostic Investigation

All patients underwent multidisciplinary developmental assessments prior to a diagnosis of a pervasive developmental disorder being established according to DSM-IV-TR criteria. Multidisciplinary assessment consisted of physical therapy, occupational therapy and/or speech pathologist assessment, in addition to assessment by a pediatrician. Need for therapist assessment was initially ascertained through a nurse-led phone-based intake interview of patients, and subsequently based on the recommendations of the pediatrician. Diagnostic formulation for all patients was made after multidisciplinary assessments, and following discussion of each patient in a multidisciplinary meeting.

CMA testing was performed using the Affymetrix Whole-Genome 2.7M Array, and breakpoints were reported using NCBI36/hg18 coordinates. Only clinically reported variants were evaluated in this study. Karyotype was performed using a standard 550 bands per haploid set Giemsa-banded method. Size of the FMR1 repeat expansion was assessed using the PCR method, and confirmed using Southern blot in affected individuals.

### 2.3. Analysis

Findings (variants) included in the clinical report for the CMA and karyotype (KT) tests were assessed and categorized into two groups. These groups were “causative” and “non-causative”. Causative results were those which were considered a likely explanation of the patient’s clinical features. Non-Causative were considered unlikely to be an adequate explanation of the patient’s clinical features.

Number needed to test (NNT) was determined by dividing the total number of participants tested by each investigation (CMA or KT) by the total number of individuals with a variant reported from the investigation (both causative and non-causative). Investigation costs were based on the full MBS rebate. Total cost was determined for each investigation type, by multiplying the number of investigations by the cost per investigation. Cost indices were developed for both all reported variants (cost per finding, CPF) and only those variants considered to be causative (cost per causative finding, CCF). CPF was determined by dividing the total cost of all investigations by the total number of reported variants. CCF was determined by dividing the total cost by the number of causative variants.

## 3. Results

Six hundred and ninety eight patients were identified with a PDD diagnosis between 17 July 2008 and 28 August 2012. Results are displayed in Table 1. One hundred and ten patients (15.7%) underwent testing with CMA, of which 12 patients (10.9%) had a variant reported. Six (5.5%) of these variants were considered to be an adequate explanation of the patient’s clinical features. One hundred and forty patients (20.0%) underwent testing with KT, of which five patients (3.5%) had a variant reported. Four (2.9%) of these variants were considered to be an adequate explanation of the patient’s clinical features. All CMA results are described in Table 2.

Two hundred and twenty one patients (31.7%) underwent testing with either CMA, KT or Fragile X testing (FRGX), 166 (23.8%) patients had been tested with either CMA or KT, and 42 patients (6.0%) had undergone testing with both CMA and KT.

The number needed to test (NNT) to identify a causative result was 15.7 for CMA, and the cost per causative test result (CCF) was AUD $10,887.25. An incremental cost benefit of CMA over KT was identified, amounting to $1,760.00 for causative variants.

**Table 1:** Yield and cost of testing in 698 patients with pervasive developmental disorder.

Test		CMACount	KTCount	FRGXCount
		(% of Requested)	(% of Requested)	(% of Requested)
Completed		110	140	167
Normal		98 (89.1)	135 (96.4)	165 (98.8)
Variant reported	All	12 (10.9)	5 (3.5)	2 (1.2)
	Causal	6 (5.5)	4 (2.9)	2 (1.2)
NNT	All	9.2	28	83.5
	Causal	18.3	35	83.5
		$	$	$
Cost Analysis	CPT	593.85	361.35	102.00
	CPF	5443.63	10,117.8	8415.00
	CCF	10,887.25	12,647.25	8415.00

CMA, chromosomal microarray; CPT, cost per test; AUD, Australian Dollar; NNT, number needed to test; CPF, cost per finding; CCF, cost per causative finding.

## 4. Discussion

Less than a quarter of patients had been investigated with chromosomal microarray in this study. As this was a retrospective review, we were unable to ascertain whether the offer of testing had been declined or the test was not offered. In practice many of these children are difficult behaviorally to venesect. In future prospective investigations of the uptake of genomic testing in clinic populations, it would be useful to ascertain which of these (or what other reason) is the predominant reason for low uptake.

Yield of testing was similar to that reported in international cohorts. Two of 12 patients had long continuous stretches of homozygosity (LCSH) reported, though the relative proportion was not reported and therefore the clinical relevance not able to be ascertained.

**Table 2:** Characteristics of the 13 patients with an abnormal chromosomal microarray.

Patient	Sex	Abnormality	Cytogenetic breakpoints	Base pairs at breakpoints	Size of Deletion	Category	Siblings	Inheritance	OMIM Reference(s)	Implicated Gene	Reference
1	M	Deletion	Xp22.32 to p22.31	5,584,212–8,337,327	2.8 Mb	causal	1 affected brother	maternal	#300427	NLGN4	[17]
2	M	Deletion	Xp22.32 to p22.31	5,584,212–8,337,327	2.8 Mb	causal	1 affected brother	maternal	#300427	NLGN4	[17]
3	M	Deletion	Xp22.33	125,959–1,963,603	1.8 Mb	unknown	unknown	unknown	-	unknown	-
4	M	Deletion	16p11.2	29,524,436–30,105,430	581 Kb	causal	unknown	de novo	#611913	unknown	-
5	M	LCSH	-	-	-	non-causal	unknown	unknown	-	-	-
6	M	Deletion	6p25.3	1,541,183–1,660,384	119 Kb	causal	non-carrier	de novo	#602884	GMDS	[18,19]
7	M	Two duplications	1q21.1; 15q11.2	144,962,948–146,296,190; 20,305,429–20,667,158	1.3 Mb; 356 Kb	unknown	unknown	unknown	-	unknown	[20,21,22]
8	M	Duplication	22q11.21	17,370,128–19,790,009	2.4 Mb	causal	unknown	paternal	#608363	unknown	[2]
9	M	Duplication	9p13.12	14,710,658–15,458,007	747 Kb	causal	unknown	unknown	-	unknown	-
10	M	Deletion	3q13.33 to 3q21.1	122,758,745–124,894,705	2.1 Mb	unknown	unknown	maternal	-	unknown	-
11	M	Deletion	Xp21.3	29,306,729–29,311,653	5 Kb	unknown	unknown	unknown	#300206; #300143	IL1RAPL1	[15,16,23]
12	M	LCSH	-	-	-	non-causal	unknown	unknown	-	-	-

LCSH, Long Continuous Stretches of Homozygosity.

### 4.1. Pre-Test Probability of Genetic Testing in Autism

Most of the guidance around testing of children with developmental delay and autism is targeted at practitioner working in a specialized context, usually a clinical genetics service. This raises the question of whether the literature, on which decision pathways have been developed, is applicable to practitioners working in a more community-orientated context.

We set out to determine whether the pre-test probability of an abnormal chromosomal microarray is less in our child development service, compared to the literature. However, community child development services also serve demographically heterogenous populations, and the question remains whether all clinic-contexts (metropolitan community, peripheral or rural pediatric services) are likely to have the same yield from genetic testing of children with autism using chromosomal microarray. Our study suggests that at least in the metropolitan context, the yield is comparable to published levels.

Determining the yield of CMA in a single state, across different service contexts (clinical genetics, developmental pediatrics, community pediatrics and rural pediatrics) would greatly assist in answering this question, with ultimate implications for children and their families, and for healthcare providers and funding-bodies.

### 4.2. Description of Clinical Service

The analysis of child development clinic populations in Australasia is complicated by the absence of a single model of service provision. Thus, the clinic population reported here might not be generalizable to other tertiary child development services.

Similarly, the diagnostic process for children with autism varies by each service. Our service assesses and diagnoses children with autism using a pragmatic approach. Structured interview and observation tools are not routinely used for our patients, and therefore the diagnostic process may not be as rigorous as the process involved in previously reported cohorts.

### 4.3. Identifying Study Population

Patients were identified by the use of our hospital billing system. Patients seen in our service are billed for their appointment with the appropriate Medicare billing code. A separate billing code is used for patients with a diagnosis of autism, however use of the Medicare billing code requires that the patient be eligible for all Medicare services, and therefore non-eligible patients were not detected by this method [23,24]. This method thereby excluded non-citizens.

### 4.4. Data Collection

Pathology tests requested through Mater Pathology, a service of Mater Health Services, are registered on the pathology information management system. However, tests requested outside of our health service, would not usually be forwarded to the Child Development Service team or Mater health record.

Cases are listed as individuals and not families: Patients 1 and 2 are siblings, one of which would be expected to be identified through cascade testing. In this case, these children were assessed and tested concurrently, and therefore both results were included.

### 4.5. Cost Analysis

The cost index analysis used is a product of the number needed to test (NNT) and the cost of the investigation. This is a highly simplistic indication of cost-utility, given that the diagnoses made are highly heterogeneous and of variable benefit to the patient and family. It does not consider, for example, the increased costs of genetic counseling and clinical review that is required because of increased yield from CMA, and because of the uncertainty of many results. A better method for providing a financial index of a test could not be found, and therefore this index was used. It may provide a useful method for comparing the total cost of investigation (but not of other aspects of patient care).

### 4.6. Reporting and Classification of Genetic Findings

Guidelines for the reporting of chromosomal microarray are yet to be published for Australasia, including which findings to include in reports. Some findings were reported for participants in the current study, which were either of uncertain meaning (variant of unknown significance) or likely non-pathogenic. Long contiguous stretches of homozygosity (LCSH) is one of these findings, for which there has been disagreement about its significance and interpretation [25], and the clinical implications remain unclear [26]. The distinction between causal and non-causal findings allowed us to distinguish between those results in which a strong case could be made for the reported variant being the underlying cause of the child’s presentation. This was assessed on a subjective basis, however one basis by which to consider variants would be the threshold of certainty at which, for instance, preimplantation genetic diagnosis (PGD) or prenatal testing (PNT) would be offered to the family. In this instance, 22q11.2 duplication for example, is a recognized duplication syndrome and cause of developmental difficulties. In contrast, LCSH may indicate consanguinity and therefore an underlying autosomal recessive condition, however does not identify single variant for which PGD or PNT could be offered.

However, with many genetic variants, there remains the possibility that a predicted pathogenic variant is not pathogenic and vice versa. An example of this from our cohort is the child with the Xp21.3 deletion. This deletion involves the intronic region of IL1RAPL1, a gene known to be associated with x-linked developmental difficulties [15,16,23]. With the currently available evidence we cannot be certain whether this is or is not the cause of this child’s developmental difficulties.

### 4.7. Learning Curve of CMA

Clinicians have had greater than forty years of use of KT in a very large number of patients, and therefore have had considerable time to assess the implication of KT in their patients. CMA has now been in use in our institution for four years with a considerably greater number of variants being detected and limited data on the implications of some of these variants. Whilst identifying pathogenic variants may be useful to the family or clinician, identifying variants of uncertain significance or non-pathogenic variants may cause distress for the family. Therefore, whilst CMA may have greater power to detect chromosomal variations, this advantage may be outweighed by the uncertain nature of many of the detected variants in the short and medium-term.

## 5. Conclusions

Though the MBS currently funds CMA for patients with developmental delay and autism, a relatively small proportion of our patient population have had this investigation performed. Therefore, the opportunity exists to identify the underlying cause of autism in a considerable number of our patients. This also raises the question of whether this is a finding in other services in Australia, and what the reasons are for this finding. CMA testing is associated with a similar yield in our population as previously reported cohorts. CMA yielded greater results and cost less “per diagnosis” than KT.
